# Treatment of Human Sporotrichosis Caused by *Sporothrix brasiliensis*

**DOI:** 10.3390/jof8010070

**Published:** 2022-01-10

**Authors:** Vanice Rodrigues Poester, Rossana Patricia Basso, David A. Stevens, Lívia Silveira Munhoz, Vanessa Brito de Souza Rabello, Rodrigo Almeida-Paes, Rosely Maria Zancopé-Oliveira, Mariza Zanchi, Jéssica Louise Benelli, Melissa Orzechowski Xavier

**Affiliations:** 1 Programa de Pós Graduação em Ciências da Saúde, Faculdade de Medicina (FAMED), Universidade Federal do Rio Grande (FURG), Rio Grande 96200-190, RS, Brazil; rpbsana@yahoo.com.br (R.P.B.); liviasmunhoz@gmail.com (L.S.M.); melissaxavierfurg@gmail.com (M.O.X.); 2 Programa de Pós Graduação em Bioquímica Toxicológica, Universidade Federal de Santa Maria (UFSM), Santa Maria 97105-900, RS, Brazil; 3 Laboratório de Micologia, FAMED, FURG, Rio Grande 96200-190, RS, Brazil; 4 Hospital Universitário-UH-FURG/Empresa Brasileira de Serviços Hospitalares—Ebserh, Rio Grande 96200-190, RS, Brazil; marizazanchi@hotmail.com; 5 California Institute for Medical Research, San Jose, CA 95128, USA; stevens@stanford.edu; 6 Division of Infectious Diseases and Geographic Medicine, Stanford University Medical School, Stanford, CA 94305, USA; 7 Laboratório de Micologia, Instituto Nacional de Infectologia Evandro Chagas, Fundação Oswaldo Cruz, Rio de Janeiro 21040-900, RJ, Brazil; vanessabritorabello@gmail.com (V.B.d.S.R.); rodrigo.paes@ini.fiocruz.br (R.A.-P.); rzancope@gmail.com (R.M.Z.-O.)

**Keywords:** *Sporothrix* spp., zoonotic sporotrichosis, itraconazole, potassium iodide, antifungal therapy

## Abstract

We describe the successful treatment of a series of 30 zoonotic sporotrichosis cases from southern Brazil. *Sporothrix brasiliensis* was the species genotypically identified in all 25 confirmed cases. Five other cases were classified as probable, without laboratory confirmation, but with clinical and epidemiological data of cat-transmitted sporotrichosis. Two isolates were sequenced by translation elongation factor-1 alpha (EF1α) loci in order to compare their sequences, and both of them showed distinct genotypes from *S. brasiliensis* strains from other Brazilian states. Itraconazole (ITZ) or potassium iodide (KI) were the first choice treatment in 28 and 2 cases, respectively. Microdilution assay showed a wild-type profile of *S. brasiliensis* isolates to ITZ. However, a lack of clinical response occurred in 42% of cases, especially those treated with ITZ 100 mg/day, and treatment needed modifications, by either increased doses or antifungal combinations. Clinical cure required a mean of 187 days of treatment, which was dependent on the clinical form of the disease and age of patients. Therapy, including dosages and durations, for cutaneous forms of sporotrichosis requires re-evaluation, since cases caused by *S. brasiliensis* may influence treatment efficacy.

## 1. Introduction

Cat to human transmission of sporotrichosis is an emerging public health problem in Brazil [1,2,3]. An unprecedented epidemic is currently ongoing in this country, with thousands of cases caused by *Sporothrix brasiliensis* in the last decade [1]. Recently, this species has been spreading inside Brazil and neighboring countries [1].

Sporotrichosis is a subcutaneous mycosis, which commonly presents as a fixed cutaneous lesion or a lymphocutaneous presentation (with lymph node commitment). Severe forms of sporotrichosis (disseminated and extracutaneous) are rare and mostly associated with immunosuppression [2,3]. Itraconazole (ITZ) and/or potassium iodide (KI) are the drugs of choice to treat cutaneous sporotrichosis, independent of etiological species. The last major guideline for sporotrichosis therapy was published in 2007 [4] and focused on the disease transmitted by plant and soil exposure, a different etiology from the disease with zoonotic transmission. Although *S. brasiliensis* infection also causes primarily cutaneous manifestations of the disease, as other species [2,3], it is the most virulent of the genus *Sporothrix* [5,6]. In addition, treatment-refractory sporotrichosis caused by *S. brasiliensis* has been reported [7]. 

Of many articles regarding cutaneous sporotrichosis treatment [2,8,9,10,11,12,13,14,15,16,17], only two focused on zoonotic transmission [2,16], and just one concerning disease caused by *S. brasiliensis* in southeast Brazil (Rio de Janeiro state; RJ, Brazil) [2]. Therefore, we describe clinical characteristics and outcome of antifungal treatment of cutaneous human sporotrichosis caused by *S. brasiliensis* in southern Brazil (Rio Grande do Sul state—RS, Brazil), a region experiencing numerous zoonotic cases, and with strains whose genotype differs from those from the RJ epidemic and other hyperendemic Brazilian states [2,3,18].

## 2. Materials and Methods

Cutaneous sporotrichosis cases diagnosed at the University Hospital of Rio Grande (UH-*Universidade Federal do Rio Grande*–FURG/*Empresa brasileira de serviços hospitalares*–Ebserh), southern Brazil, between 2017 and 2020 were included. The patients had classical lesions, most had *Sporothrix* spp. isolation in culture and an epidemiological history of zoonotic transmission. Isolates were identified by molecular methods (Polymerase Chain Reaction—PCR species-specific) [19] using the primers SbraF (5′-CCC CCG TTT GAC GCT TGG) and SbraR (5′-CCC GGA TAA CCG TGT GTC ATA AT). Only patients with sporotrichosis caused by *S. brasiliensis* were included in the study. In addition, two isolates (GenBank identification: OL853841 and OL853842) were sequenced by translation elongation factor-1 alpha (EF1α) loci in order to compare the RS isolates sequences from RJ and other Brazilian states, following the protocol described by Rodrigues et al. [18]. The automated sequencing was conducted at the Fiocruz Technological Platforms Network. The sequences were edited by Sequencher Software Package (version 4.9, Ann Arbor, MI, USA). Phylogenetic analyses were carried out using Maximum Likelihood by Molecular Evolutionary Genetics Analysis (MEGA) 6 software (Arizona State University, Tempe, AZ, USA). Sequences of *Sporothrix* spp. deposited in the GenBank were included in this analysis (Table 1).

Patients were followed from the diagnosis until clinical cure, via monthly outpatient visits. The treatment (antifungal, dosages) for adults and children followed the international protocol described by Kauffman et al. [4]. Patients who abandoned treatment during the follow-up were excluded from the analysis. Photographic records, clinical, and epidemiological data were collected. Data regarding sex, age, diagnosis, clinical presentation, antifungal therapy, and adverse effects were analyzed. The total time of the treatment duration was compared between patients with fixed cutaneous sporotrichosis and lymphocutaneous sporotrichosis using the Student’s t-test from the statistical program SPSS 25.0 (IBM^®^, Armonk, NY, USA).

In vitro susceptibility tests were performed with available isolates to determine the minimal inhibitory concentration (MIC) to ITZ, defined as the lowest concentration that inhibited 100% of the fungal growth, following the Clinical and Laboratory Standards Institute (CLSI M38-ED3) guideline [20]. ITZ was tested in concentrations ranging from 0.125 to 8 µg/mL. The isolates were classified as ITZ wild-type (MIC ˂ 2 µg/mL) or ITZ non-wild type (MIC ≥ 2 µg/mL) and the geometric mean (GM) was calculated [21].

## 3. Results

During this period, 46 patients were diagnosed with cutaneous sporotrichosis. One patient was infected by *S. schenckii* and was excluded from the study. Fifteen patients abandoned treatment during the follow-up. Twenty-five patients had *S. brasiliensis* isolation from a lesion culture, and five were diagnosed by clinical presentation and compatible epidemiologic history. 

**Figure 1:**
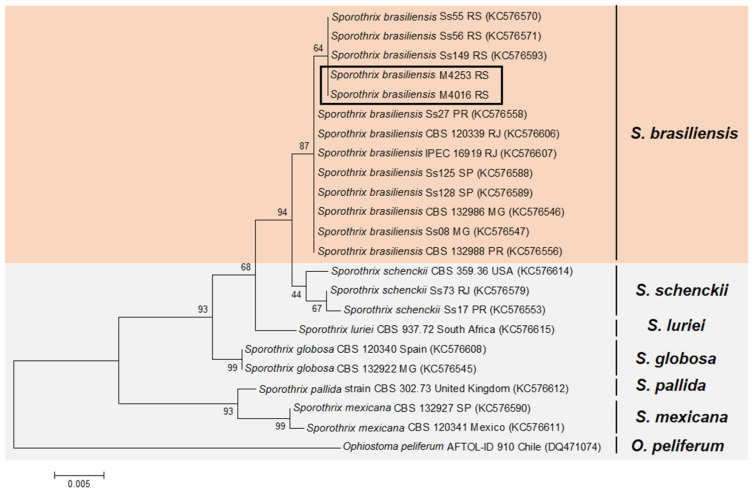
Genotyping of *Sporothrix brasiliensis* strains from Rio Grande do Sul. Maximum likelihood method based on partial sequences of the translation elongation factor-1 alpha (EF1a). A bootstrap value of 1000 is represented in the branches. Orange marked: *S. brasiliensis* strains; Gray marked: other *Sporothrix* species. Rectangle: Isolates included in our study.

The comparison of the genotypes showed that the two strains included in our study from Rio Grande do Sul were clustered in a different clade from *S. brasiliensis* strains from Rio de Janeiro, São Paulo, Minas Gerais and Paraná (Figure 1). 

Most patients were women (63%; *n* = 19) and adults (87%; *n* = 26), mean of 48 years old (range 18–89). Four pediatric cases occurred (13%), ages 2–9 years (mean 7 years old). None had an immunocompromising co-morbidity. Scratch and/or bite by infected cats was reported by 43% (*n* = 13) of the patients, and only contact with injured cats was reported by 53% (*n* = 16). The mean onset of disease until diagnosis was 80 days (range 3–540). 

During the follow-up, 42% (11/30) of the patients had therapeutic failure, and their therapeutics was modified after 30–175 days (mean of 91 days) after beginning treatment, by increasing ITZ dosage and/or by adding KI (5 to 50 drops/3 times daily).

### 3.1. Adult Cases

In the adult cases, there was a predominance of the lymphocutaneous form (62%; 16/26), the remainder were focal skin lesions (38%; 10/26). Lesions were commonly localized on the arms (85%; 22/26). Facial or leg lesions occurred in two patients each. 

**Figure 2:**
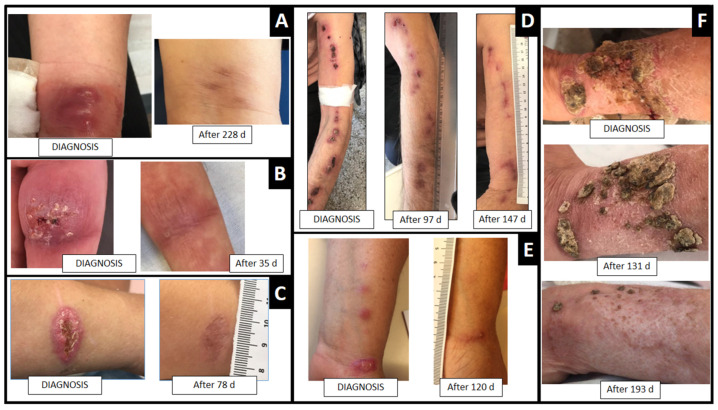
Clinical cure in days (d) of adult patients with cutaneous sporotrichosis from southern Brazil treated with ITZ and/or KI. (**A**,**B**,**D**,**E**). Lymphocutaneous form of sporotrichosis localized in the upper limb. (**C**,**F**). Focal cutaneous form of sporotrichosis localized to the upper limb.

ITZ was the first-choice drug used in 96% (*n* = 25) of cases in daily doses of 100–400 mg, one (4%) was referred with KI therapy (20 drops/3 times daily) already initiated. All patients received ≥30 days therapy after clinical cure, as consolidation therapy (Figure 2).

Among ten patients (10/26) with fixed cutaneous sporotrichosis, ITZ 100 mg/day or 200 mg/day was the first therapeutic choice in 3 and 7 patients, respectively. An increase in ITZ dosage to 200 mg/day was necessary in all patients receiving 100 mg/day, due to an unsatisfactory clinical response. Regarding patients who started therapy with ITZ 200 mg daily, an increase to 400 mg/day was necessary in 14% (1/7). 

In contrast, 94% (15/16) of patients with the lymphocutaneous form were initially treated with ITZ 200 mg/day, one patient was the one referred with KI therapy. Of the 15 cases started on ITZ, in 47% (*n* = 7) ITZ dosage needed to be increased to 400 mg/day (*n* = 4), and/or KI needed to be added (*n* = 3), to achieve a satisfactory clinical response. 

Clinical cure of the fixed cutaneous (*n* = 10) and lymphocutaneous (*n* = 16) occurred after an average of 148 days (range 51–292) and 218 days (range 78–361) of treatment (*p* = 0.09), respectively. Possible adverse effects associated with antifungal therapy were observed in 23% (6/26) of the adult patients, after an average of 27 days of treatment. Increase in hepatic enzymes was associated with ITZ therapy in two patients, and paresthesia or polyarthralgia in one each. Thyroid suppression was associated with KI therapy (*n* = 2).

### 3.2. Pediatric Cases

**Figure 3:**
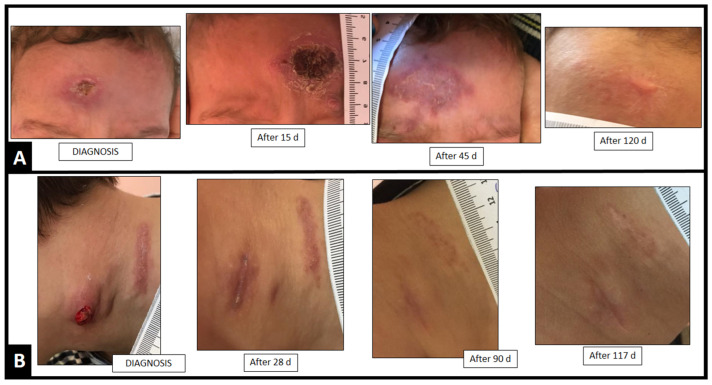
Clinical cure in days (d) of pediatric patients with cutaneous sporotrichosis from southern Brazil treated with ITZ and/or KI. Lymphocutaneous cases of sporotrichosis localized to face (**A**) and in neck (**B**).

Concerning the four pediatric cases, all had the lymphocutaneous form, localized to the face (*n* = 3) or neck (*n* = 1). ITZ (100–200 mg daily) was the first therapeutic choice in 3, and in one therapy with KI (2 g/daily) was initiated before referral to the study center. Two children had complete remission of the lesions from their initial ITZ treatment, without adverse effects. KI was added to ITZ in one child without clinical response after 14 days of ITZ 100 mg/day. The other child showed adverse effects from KI (diarrhea and thyroid suppression, after 7 and 14 days of therapy, respectively), and the therapy was modified to ITZ 200 mg daily. Clinical cure occurred after a mean of 114 days (range, 90–146) of treatment (Figure 3).

The ITZ tests in vitro showed that all isolates (*n* = 23) had susceptibilities consistent with studies on wild type isolates, with MIC values ranging from ≤0.125 to 1 µg/mL, and a geometric mean of 0.33 µg/mL.

## 4. Discussion

Our study expands the data regarding treatment of human zoonotic cases of sporotrichosis in Brazil caused by *S. brasiliensis* [2]. This is the first study on this topic from the southern Brazilian region. [2]. Cases from southern RS, Brazil, are predominantly zoonotic [3]. Our patients were mostly women with the lymphocutaneous form, and a few pediatric cases with predominance of disease on the face, consistent with other reports [2,3]. 

ITZ (100–400 mg/day) is the drug of choice for sporotrichosis treatment, leading to a clinical cure in almost all patients, with low rates (10–40%) of adverse effects [2,13,15,16]. Our series is partially in agreement with these data, showing clinical resolution of the lesions through therapy with ITZ, and a rate of ~25% adverse effects. However, the duration of the treatment needed was longer in our study (27 weeks average) in comparison with others described (average, 12–24 weeks) [2,13,15,16]. In addition, ITZ 100 mg/day was insufficient to cure patients, in contrast with patients from RJ treated with this antifungal dose [2].

This longer period of antifungal therapy may be related, at least in part, to a delayed diagnosis in our region, as the average time between the appearance of the lesions and the patients’ first appointment was almost three months (mean, 11 weeks). Consequently, lymphocutaneous lesions predominated in our patients, requiring a mean period of treatment of 10 weeks longer than for the fixed cutaneous form. The public in our region must be educated about sporotrichosis, to facilitate an early diagnosis, which is conducive to a better prognosis [2].

Although no in vitro resistance to ITZ was detected, in almost half of our patients, mostly with the lymphocutaneous manifestation and in patients receiving a low dose of ITZ (100 mg/day), the therapy needed to be changed due to a lack of clinical response. These data of the lack of clinical response contrast that described for the treatment of sapronotic cases, which are frequently cured with only KI [9,10] and to the zoonotic cases from RJ, where ITZ 100 mg daily was sufficient to cure 85% of patients [2]. In our study only one pediatric patient was cured with this low a dose. 

One limitation of our study was the few isolates that could be included in the genotypic analysis. However, our results are in agreement with another study of *S. brasiliensis* genotyping [18], suggesting that the RS isolates are genotypically different from those from São Paulo, Minas Gerais, Paraná, and RJ, which has the largest case series of zoonotic sporotrichosis treatment in Brazil [2].

Although the dosages and therapeutic regimen recommended by the international guidelines for sporotrichosis were effective in our study [4,22], the search for new drugs for the treatment of this *S. brasiliensis* problem are urgent, considering the few options of antifungals available [23,24,25]. Indeed, the high virulence of *S. brasiliensis* [5] enforces the need for a protocol emphasizing Brazilian data, which is the global epicenter of sporotrichosis caused by this species [1]. Genotyping difference of the RS strains in comparison with strains from other Brazilian states, and its impact on virulence and/or antifungal susceptibility, deserves to be investigated further.

## Figures and Tables

**Table 1:** Isolates and GenBank Identification.

Isolate Code	Species	Geographic Origin	GenBank EF1α
CBS 120339	*S. brasiliensis*	Rio de Janeiro, RJ, Brazil	KC576606
IPEC 16919	*S. brasiliensis*	Rio de Janeiro, RJ, Brazil	KC576607
Ss55	*S. brasiliensis*	Rio Grande, RS, Brazil	KC576570
Ss56	*S. brasiliensis*	Rio Grande, RS, Brazil	KC576571
Ss149	*S. brasiliensis*	Pelotas, RS, Brazil	KC576593
M4016	*S. brasiliensis*	Rio Grande, RS, Brazil	OL853841
M4253	*S. brasiliensis*	Rio Grande, RS, Brazil	OL853842
CBS 132988	*S. brasiliensis*	Curitiba, PR, Brazil	KC576556
Ss27	*S. brasiliensis*	Curitiba, PR, Brazil	KC576558
Ss125	*S. brasiliensis*	Campinas, SP, Brazil	KC576588
Ss128	*S. brasiliensis*	Campinas, SP, Brazil	KC576589
CBS 132986	*S. brasiliensis*	Belo Horizonte, MG, Brazil	KC576546
Ss08	*S. brasiliensis*	Belo Horizonte, MG, Brazil	KC576547
CBS 359.36	*S. schenckii*	United States of America	KC576614
Ss73	*S. schenckii*	Rio de Janeiro, RJ, Brazil	KC576579
Ss17	*S. schenckii*	Curitiba, PR, Brazil	KC576553
CBS 120340	*S. globosa*	Spain	KC576608
CBS 132922	*S. globosa*	Belo Horizonte, MG, Brazil	KC576545
CBS 120341	*S. mexicana*	Mexico	KC576611
CBS 132927	*S. mexicana*	São Paulo, SP, Brazil	KC576590
CBS 937.72	*S. luriei*	South Africa	KC576615
CBS 302.73	*S. pallida*	United Kingdom	KC576612
AFTOL-ID 910	*Ophiostoma piliferum*	Chile	DQ471074

RJ: Rio de Janeiro; RS: Rio Grande do Sul; PR: Paraná; SP: São Paulo; MG: Minas Gerais.

## Data Availability

Not applicable.
